# Noise-induced and age-related hearing loss:  new perspectives and potential therapies

**DOI:** 10.12688/f1000research.11310.1

**Published:** 2017-06-16

**Authors:** M Charles Liberman

**Affiliations:** 1 Department of Otolaryngology, Harvard Medical School, Eaton Peabody Laboratories, Massachusetts Eye and Ear, 243 Charles St., Boston, MA, 02114, USA

**Keywords:** sensorineural hearing loss, noise-induced hearing loss, auditory neurons

## Abstract

The classic view of sensorineural hearing loss has been that the primary damage targets are hair cells and that auditory nerve loss is typically secondary to hair cell degeneration. Recent work has challenged that view. In noise-induced hearing loss, exposures causing only reversible threshold shifts (and no hair cell loss) nevertheless cause permanent loss of >50% of the synaptic connections between hair cells and the auditory nerve. Similarly, in age-related hearing loss, degeneration of cochlear synapses precedes both hair cell loss and threshold elevation. This primary neural degeneration has remained a “hidden hearing loss” for two reasons: 1) the neuronal cell bodies survive for years despite loss of synaptic connection with hair cells, and 2) the degeneration is selective for auditory nerve fibers with high thresholds. Although not required for threshold detection when quiet, these high-threshold fibers are critical for hearing in noisy environments. Research suggests that primary neural degeneration is an important contributor to the perceptual handicap in sensorineural hearing loss, and it may be key to the generation of tinnitus and other associated perceptual anomalies. In cases where the hair cells survive, neurotrophin therapies can elicit neurite outgrowth from surviving auditory neurons and re-establishment of their peripheral synapses; thus, treatments may be on the horizon.

## Introduction

According to the Centers for Disease Control, 25% of American adults suffer from some form of noise-induced hearing loss (NIHL). Our ears were not designed to withstand long and repeated exposure to the high sound pressures produced by the machinery that surrounds us in modern industrialized society, be it work-, leisure- or combat-related. Correspondingly, with increasing life expectancy, the prevalence of age-related hearing loss (AHL) is also on the rise. The National Institute on Deafness estimates that 33% of people over the age of 65 have significant hearing impairment. The two types of hearing loss are likely interrelated, as people in minimally industrialized areas (e.g. the Sudanese desert) do not show the inexorable age-related deterioration of hearing seen in the developed world ^1^.

**Figure 1.**
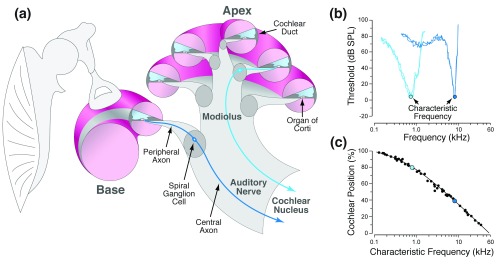
Mapping of characteristic frequency along the cochlear spiral. **(a)** Schematic showing middle ear bones and a cross-section through the cochlear spiral, illustrating perilymph (pink) and endolymph spaces (blue) and two auditory nerve fibers (ANFs), one high-frequency (deep blue) and one low-frequency (cyan), traveling from organ of Corti through the modiolus to the cochlear nucleus. **(b)** Tuning curves for a high- and a low-frequency ANF, showing threshold as a function of frequency. The characteristic frequency ^48^ defines where the fiber originates along the mechanically tuned cochlear spiral. **(c)** Cochlear frequency map derived from intracellular labeling in the cat defines the precise relationship between characteristic frequency and cochlear location ^76^. dB SPL, decibels sound pressure level.

Both NIHL and AHL are known as sensorineural hearing loss because the dysfunction arises in the inner ear, or cochlea, where sound-induced vibrations are transduced by sensory hair cells into electrical signals in cochlear neurons that relay the encoded information to the brain ( Figure 1). For decades, we've known that hair cell damage is a key contributor to the hearing loss in NIHL and AHL ^2– 4^, as defined by the audiogram, which measures the minimal sound pressure required for pure-tone detection in a quiet test booth. For decades, it was assumed that cochlear neural loss occurred only after hair cell death ^5^ and thus was rarely of functional significance in NIHL or AHL.

Recently, my lab showed, in both NIHL and AHL, that synaptic connections between hair cells and cochlear neurons can be destroyed well before the hair cells are damaged ^6^. This synaptic loss silences large numbers of cochlear neurons but is invisible in routine histological material and does not affect tests of threshold detection, so long as the loss is not complete. This cochlear synaptopathy, also known as "hidden hearing loss", compromises performance on difficult listening tasks such as understanding speech in a noisy environment, which is the classic complaint of those with NIHL and AHL. In animal models, post-exposure treatment with neurotrophins, delivered locally to the inner ear, can repair or replace the damaged synapses ^7^, suggesting possible future therapies for some of the most disabling sensory impairments in sensorineural hearing loss.

## Normal cochlear function

**Figure 2.**
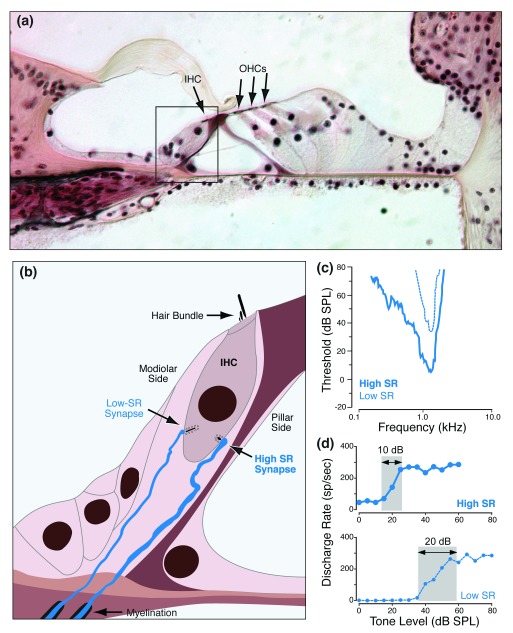
High- vs. low-SR auditory nerve fibers and their synaptic localization on the inner hair cell. **(a)** Light micrograph of the organ of Corti, as it appears in conventional histological material, stained with hematoxylin and eosin. Peripheral terminals of auditory nerve fibers (ANFs) in the inner hair cell (IHC) area (box) are not resolvable. **(b)** Schematic of type I peripheral terminals showing that fibers with high versus low spontaneous discharge rates (SRs) make synaptic contacts on opposite sides of the IHC. **(c)** High-SR fibers have lower thresholds than do low-SR fibers, as shown by these two tuning curves. **(d)** High-SR fibers have smaller dynamic ranges (grey box) than do low-SR fibers when stimulated with tone bursts at the characteristic frequency. dB SPL, decibels sound pressure level; OHC, outer hair cell.

The mammalian cochlea is a spiraling, fluid-filled tube within a particularly dense bone ( Figure 1a). In cross-section, the spiraling bony tube is bisected by a membranous tube called the cochlear duct, the lumen of which is lined with epithelial cells, including three rows of outer hair cells (OHCs) and one row of inner hair cells (IHCs) ( Figure 2a). Each hair cell has, at its lumenal end, a “hair bundle", i.e. a tuft of modified microvilli, called stereocilia, where the mechanoelectrical transduction channels are found. Sound-evoked vibration of the sensory epithelium opens these channels, causing hair cell depolarization and release of neurotransmitter (glutamate) from the other end of the hair cell, where synapses with auditory nerve fibers (ANFs) are located ( Figure 2b). The entire spiraling sensory epithelium is mechanically tuned and is most responsive to high frequencies at the "basal" end, i.e. closer to the stapes, and to low frequencies at the "apical" end ( Figure 1b and c).

**Figure 3.**
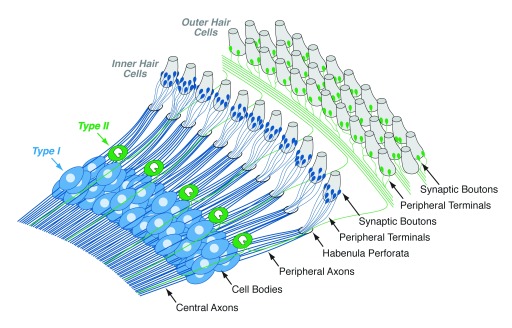
Innervation patterns of type I and type II auditory nerve fibers on inner and outer hair cells, respectively. Central and peripheral axons of type I cells are myelinated, whereas axons of type II neurons are unmyelinated. Peripheral terminals of type I and type II cells are unmyelinated within the organ of Corti, i.e. beyond the habenula perforata.

**Figure 4.**
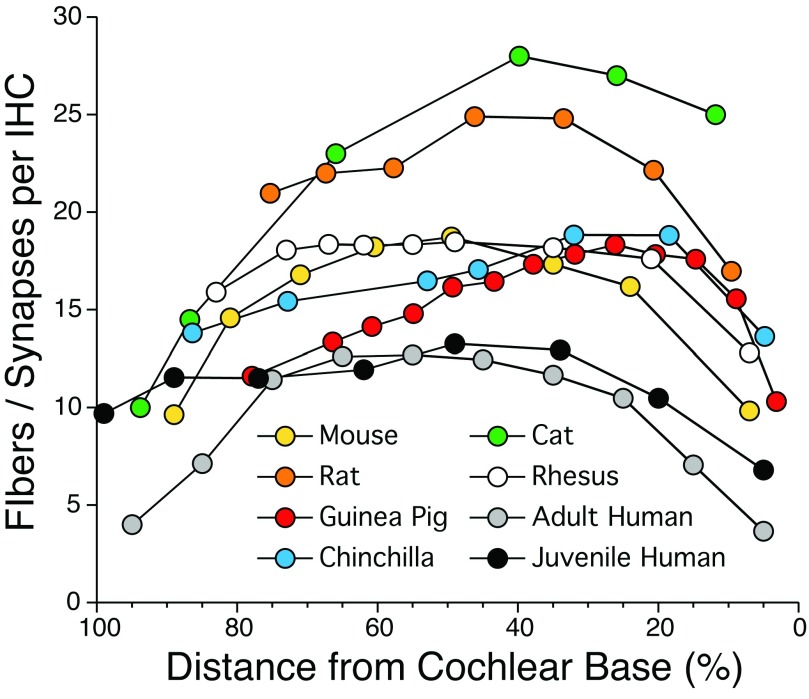
Normal density of auditory nerve fibers along the cochlear spiral. Data from the mouse, rat, guinea pig, chinchilla, rhesus monkey, and adult human are from the Liberman lab and are based on confocal analysis of immunostained synapses from cochlear epithelial whole mounts such as in Figure 5. Cat data are from a serial-section ultrastructural study ^77^. Data from juvenile human are based on light-microscopic counts of peripheral axons from a 7-year-old ^42^. Deviation between the two sets of human data at low frequencies may arise because ANFs in apical cochlear regions often form two synapses each ^11^.

In humans, the cochlear spiral is ~32 mm long and contains roughly 3,200 IHCs and 10,000 OHCs ^8^. The two hair cell types have different functions. The OHCs have been called the "cochlear amplifier" because they possess electromotility, which is driven by molecular motors containing a membrane protein called prestin ^9^. Prestin undergoes a voltage-sensitive conformational change that turns sound-driven hair-cell receptor potentials back into mechanical motion that is powerful enough to vibrate the entire sensory epithelium, including the IHC stereocilia. The IHCs are more conventional sensory receptors, generating the pre-synaptic drive for all the myelinated sensory fibers of the auditory nerve. Each ANF has a bipolar "type I" cell body in the spiral ganglion that sends a myelinated peripheral axon towards the sensory epithelium, where its unmyelinated terminal contacts a single IHC, and a myelinated central axon to the cochlear nucleus ( Figure 3), the first central processing station in the ascending auditory pathway ^10^. In humans, as shown in Figure 4, each IHC is contacted by 4–13 ANFs ^11, 12^ depending on cochlear location; thus, each auditory nerve contains ~40,000 myelinated sensory fibers. The OHCs are contacted by a much smaller population (5–10%) of thin, unmyelinated fibers ^13^. These "type II" ANFs also project to the cochlear nucleus ^14^. Their function is unclear, but they may be nociceptors ^15– 17^.

**Figure 5.**
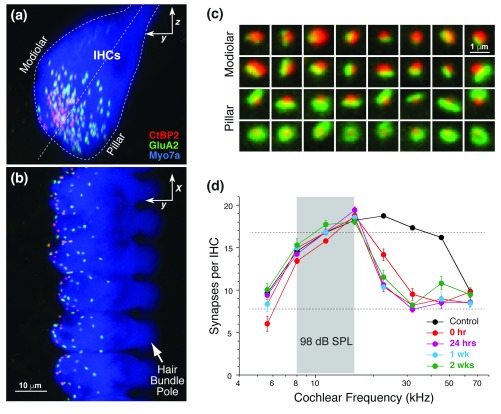
Noise-induced cochlear synaptopathy in the mouse. ( **a**, **b**) Confocal images of mouse inner hair cells (myosin 7a – blue) immunostained for pre- and post-synaptic markers (CtBP2 – red, GluA2 – green) to reveal the synaptic contacts. **Panel (b)** shows the maximum projection of a focal series through six adjacent inner hair cells; **Panel (a)** shows the same image stack projected into the orthogonal plane to show a cross-sectional view like that schematized in Figure 2a. ( **c**) High-power views of 32 synaptic puncta, segregated according to position on the inner hair cell (IHC) (modiolar versus pillar, see Figure 2a): low spontaneous discharge rate (SR) synapses are found on the modiolar side and have larger ribbons and smaller glutamate receptor patches. ( **d**) Synaptic counts on inner hair cells from noise-exposed ears at several post-exposure times (from 38). The exposure (octave band noise at 98 decibels sound pressure level [dB SPL] for 2 hours) produced only a transient threshold elevation and no loss of hair cells.

## Hearing loss and hair cell damage in AHL and NIHL

As for most types of hearing dysfunction, characterizing NIHL and AHL in humans begins with the threshold audiogram, which measures the lowest audible sound pressure for pure tones at octave-frequency intervals, typically 0.25, 0.5, 1, 2, 4, and 8 kHz (middle C is ~0.25 kHz, and the highest note on the piano is ~4 kHz). Human hearing is normally most sensitive near 1 kHz, where average sound pressure at threshold in young adults is 2 × 10 ^–5^ newtons/m ^2^, which is defined as 0 dB SPL (decibels sound pressure level). The dB scale is logarithmic, and each 20 dB increment corresponds to a 10-fold increase in the amplitude of the sound wave. The ear has an enormous dynamic range: loudness grows monotonically over at least a 100 dB range (10 ^5^ × threshold pressure) and the threshold of pain is cited as 140 dB SPL (10 ^7^ × threshold pressure) ^18^.

Cross-sectional studies in the 1960s documented the rise in audiometric thresholds with increasing years of exposure in noisy factories ^19^, where, prior to federal regulation of workplace noise, SPLs were in excess of 100 dB SPL (current regulations limit an 8-hour workday exposure to 85 dB SPL A-weighted). In its early stages, NIHL is often seen as a "notch" (i.e. threshold elevation) in the audiogram at 4 kHz. As exposure-time accumulates, the hearing loss extends to 8 kHz, and ultimately the audiogram can reveal no hearing sensation above 1 or 2 kHz, even at the highest sound pressures tested. AHL, as documented in cross-sectional studies, also affects the high frequencies first and can often lead to high-frequency deafness similar to that in advanced NIHL ^20^.

At extremely high sound pressures, such as in blast injury (>180 dB SPL peak), there can be eardrum rupture and disarticulation of the ossicles ^21^. However, for continuous-noise exposures at sound pressures like those in even the noisiest pre-regulation factories, damage is restricted to the inner ear, and OHCs are particularly vulnerable ^2^. Complete loss of OHCs will elevate thresholds by 40–60 dB ^22^. Loss of IHCs will silence all sound-evoked activity from affected cochlear regions ^2^. Many surviving IHCs and OHCs suffer stereocilia damage that compromises function and can produce larger threshold shifts than predicted by the number of lost hair cells alone ^23^. In animal studies, a single 2-hour exposure at 115 dB SPL can destroy all IHCs and OHCs throughout the basal (high-frequency) half of the cochlear spiral ^2, 24^. Fortunately, humans may be somewhat less vulnerable to noise than are smaller mammals, requiring higher SPLs or longer exposures to produce comparable damage ^25^. Nevertheless, human ears with advanced NIHL or AHL also show extensive hair cell loss throughout the high-frequency cochlear regions ^26^.

## Cochlear synaptopathy in NIHL and AHL

Although hair cell damage and death can be seen in minutes to hours after acoustic overexposure, death of spiral ganglion cells (the cell bodies of ANFs) is delayed by months to years ^27^. This observation led to the dogma that hair cells are the primary target of noise damage and that neurons die only secondarily to loss of their peripheral synapses ^5^. It has been known since the early 1980s that noise can lead to severe swelling of ANF terminals at their IHC synapses when examined within 24 hours post exposure ^28, 29^. This swelling, often accompanied by membrane rupture and loss of cytoplasmic contents, appears to be a kind of glutamate excitotoxicity, as it can be mimicked by cochlear perfusion of glutamate agonists and partially blocked by perfusion of glutamate antagonists ^30, 31^. However, ANF terminal swelling can be observed in ears with temporary threshold shifts (TTSs), and it disappears within a few days as thresholds recover. This threshold recovery led to the idea that neural connections recover or regenerate after noise damage, so long as the hair cells survive ^32^.

However, for years, no one counted ANF terminals in recovered ears because these unmyelinated endings and their synaptic connections are invisible in routine light-microscopic material ( Figure 2a), and the serial-section ultrastructural analysis required to count them is extremely labor intensive ^33^. Furthermore, threshold recovery, *per se*, is not proof of synaptic recovery. Cochlear function in these animal experiments is measured by recording ensemble ANF activity in response to brief tone bursts via metal electrodes on the cochlear capsule (compound action potential [CAP]) or in the skin of the external ear (auditory brainstem responses [ABRs]). Thresholds for these "gross" neural potentials are very sensitive to OHC damage, which can severely attenuate sound-evoked cochlear vibrations, but extremely insensitive to subtotal neural degeneration. This is because loss of ANFs and their contributions to the ensemble response can be readily compensated for, especially near threshold, by small increases in stimulus level, which recruit more responding fibers by spreading sound-evoked vibrations farther along the mechanically tuned cochlear spiral.

Thus, for many years, the question of whether or not noise destroys ANF synapses on surviving IHCs was not pursued. Then, my colleague Sharon Kujawa asked me to collaborate on a study of the interaction between NIHL and AHL. She exposed mice as young adults to a noise designed to produce a modest (40 dB) permanent threshold shift (PTS) and then let them age for 2 years to see if the cochlea deteriorated more rapidly in exposed versus unexposed animals. No prior work, to our knowledge, had followed animals for so long post exposure. Two years later, the noise-exposed mice showed ~50% loss of spiral ganglion cells in the basal half of the cochlea versus <5% in age-matched controls despite no significant loss of IHCs or OHCs in either group ^34^. Thinking back to the work on acute noise-induced ANF terminal swelling, we speculated that the exposure in our mice might be causing immediate and irreversible synaptic damage, which was revealed only by the extremely slow death of the disconnected spiral ganglion cells.

To pursue the question, we modified published immunostaining protocols to allow rapid quantification of ANF synaptic contacts in the light microscope ^35^. Each ANF contacts a single IHC via a single terminal bouton ( Figure 2 and Figure 3), forming a synaptic plaque containing (typically) a single pre-synaptic ribbon ^33, 36^. Thus, cochleae immunostained for a ribbon protein (CtBP2, red) and a glutamate receptor subtype (GluA2, 3, or 4, green) show pairs of closely apposed red and green, pre- and post-synaptic puncta ( Figure 5). Counts of puncta pairs from images acquired with confocal microscopy closely match values for ANF/IHC synapses in mice seen in a serial section ultrastructural analysis ^36^ and thus provide a rapid and robust measure of synaptic integrity in the IHC area. Each IHC is contacted by 4–28 ANFs depending on the species and cochlear location ( Figure 4): although humans have fewer ANFs per IHC than do smaller mammals, the number of ANFs per cochlea is greater because our cochlea is much longer and has many more IHCs.

We now know that even exposures producing only a TTS, and leaving all hair cells intact, can destroy up to 50% of IHC synapses across large cochlear regions ( Figure 5D). The damage is seen at cochlear regions tuned to frequencies higher than the exposure band because cochlear mechanics are non-linear: the region maximally stimulated at low SPLs (which defines “cochlear frequency”) is apical to the region maximally stimulated at high SPLs ^37^. The synaptic loss appears immediately after the noise ^38^ and, in the mouse, only worsens with increasing post-exposure time ^39^. In guinea pigs, there is partial post-exposure recovery of synaptic counts, but this may represent transient down- and up-regulation of ribbon or receptor proteins rather than degeneration and regeneration of synaptic contacts. This widespread synaptic loss in the absence of significant hair cell loss has been replicated in noise-exposed rats, guinea pigs, chinchillas, and monkeys (for review, see 40). Synaptopathy also appears in ears exposed to ototoxic drugs such as aminoglycoside antibiotics; significant loss of IHC synapses appears at doses below those causing hair cell loss or threshold shifts ^41^. Synaptopathy also appears in AHL: aging mice show synaptic loss before OHC loss (and the associated threshold shifts) ^41^, and surviving IHCs at the end of the mouse lifespan have lost ~50% of ANF synapses ^38, 41^. Normal-aging humans, i.e. those without explicit otologic disease, can also show dramatic cochlear neuropathy in regions of minimal hair cell loss ^42^: e.g. one 89-year-old ear retained only ~20% of the normal complement of ANF contacts despite minimal loss of either IHCs or OHCs ^12^.

The mechanisms underlying noise-induced synaptic damage have not been clarified beyond the cochlear perfusion studies of glutamate excitotoxicity in the 1980s ^30, 31^. Recent work showing that synaptopathy also occurs after a single high-intensity shockwave ^43^ suggests that prolonged overexposure of the post-synaptic membrane to glutamate may not be required. Furthermore, it is unclear whether noise-induced, age-related, and drug-induced synaptopathy all share the same mechanism.

## Hidden hearing loss and problems hearing in noise

Regardless of underlying mechanisms, emerging evidence suggests that surviving IHCs are partly or largely disconnected from their primary sensory fibers in many types of acquired sensorineural hearing loss. This synaptopathy has been called "hidden hearing loss" ^44^ because the damage is not visible in routine cochlear histopathology and because primary neural degeneration does not significantly affect the threshold audiogram until it exceeds ~80% ^45, 46^. Although not needed for pure-tone detection in quiet environments, a full complement of ANFs is likely required for more difficult listening tasks.

Recordings from single ANFs in normal and noise-exposed animals suggest how synaptopathy might especially compromise hearing in noisy environments. In the normal ear, ANFs comprise at least two subgroups: low-threshold fibers with high spontaneous discharge rates (SRs) and high-threshold fibers with low SRs ( Figure 2c), constituting ~60% and 40% of the ANF population, respectively ^47– 49^. Although both high- and low-SR fibers can contact the same IHC ( Figure 2b), their synapses are spatially segregated around the IHC circumference ( Figure 2 and Figure 5) and their central projections are different ^50– 52^. Their sensitivity differences likely arise from a combination of pre- and post-synaptic differences in channel expression and input resistance, respectively ^53, 54^. Single-fiber recording studies have shown that low-SR synapses are the first to degenerate in AHL ^55^, NIHL ^56^, and at least one kind of drug ototoxicity ^57^. The reasons for their heightened vulnerability are not clear but may be related to the paucity of mitochondria in their peripheral terminals ^33^, as mitochondria, in supplying ATP for Ca ^2+^ pumps, are critical to the regulation of intracellular Ca ^2+^, and Ca ^2+^ overload is critical in the genesis of glutamate excitotoxicity ^58^. Persistent abnormalities in some high-SR responses have also been reported in synaptopathic guinea pigs ^59^.

As shown in Figure 2d, the high-threshold, low-SR fibers normally extend the dynamic range of the auditory periphery ^60, 61^, but their loss should not affect threshold detection of stimuli in an otherwise quiet environment. In the presence of continuous masking noise, however, their contributions become more critical, and their loss becomes more handicapping. By virtue of their higher thresholds, low-SR fibers are more resistant to "masking" by continuous noise ^62^. As the noise level rises, low-threshold, high-SR fibers are driven to "saturated" discharge rate, leaving only the high-threshold, low-SR fibers to carry information about stimuli embedded in the noise.

## Hidden hearing loss in humans: diagnosis and treatment

Difficulty hearing in noise is a major complaint of people with sensorineural hearing loss, and it has long been known that two people with the same audiogram, whether normal or abnormal, can perform differently on speech-in-noise tests. Prior to the discovery of hidden hearing loss, these differences were ascribed largely to differences in central auditory processing. A few human histopathological studies suggest that cochlear synaptopathy is an important component of human sensorineural hearing loss, and one even suggests that it is correlated with word-recognition scores ^63^. However, the inner ear cannot be biopsied, so enhanced diagnostic tests are needed to screen living subjects.

In mouse studies, we showed that suprathreshold amplitudes of ABR wave 1, the summed onset responses of ANFs, were well correlated with the degree of cochlear synaptopathy, so long as cochlear sensitivity was not compromised due to OHC dysfunction ^6, 41^. Once thresholds are elevated, it is difficult to separate changes due to synaptopathy from those due to hair-cell damage. Auditory evoked potentials such as ABRs are easily measured in human subjects from scalp and/or ear-canal electrodes. In a recent study of young adults with normal audiograms, we found a correlation between performance on a difficult speech-in-noise test and alterations in auditory evoked potentials that were consistent with cochlear synaptopathy ^64^. Having purposely sought out subjects who abused their ears (aspiring musicians who never wore ear protection) and those who routinely protected their ears, we also noted a correlation between ear abuse and poorer performance on speech-in-noise tests. Other studies have shown correlations among normal-threshold young subjects between the ability to perform complex listening tasks and alterations in ABRs that suggest a peripheral rather than a central origin ^65^. A recent study of military veterans with normal audiograms has also found a correlation between ABR wave 1 amplitudes and noise-exposure history ^66^, while another recent study of "normal-hearing" subjects in the UK failed to find such a correlation ^67^. However, different metrics of noise history were used, and neither study correlated the electrophysiological results with performance on speech-in-noise tasks.

Clearly, more work is needed in this area. However, existing data from humans and animals make it clear that significant cochlear neural damage can occur without hair cell damage and thus can hide behind a normal audiogram. This neural damage is likely to be a handicap in difficult listening situations, especially as overt hearing loss (i.e. threshold elevation and hair cell damage) is added to the mix. Since existing federal guidelines on workplace noise exposure were derived based on the assumption that exposures producing no PTSs are benign ^68^, a careful re-evaluation of these guidelines is warranted if hidden hearing loss is to be prevented as well.

An exciting aspect of this work is the notion that some of the hearing handicap in sensorineural hearing loss might be treatable or preventable ^69^. In mammalian cochleae, including those in humans, hair cells and cochlear neurons are post-mitotic, and damaged or lost elements are never replaced ^70^. Although limited hair cell regeneration via transdifferentiation of remaining support cells has been demonstrated in animal models ^71^, the repair of cochlear synaptopathy is arguably simpler because there is an extended therapeutic window in which the hair cell targets as well as the spiral ganglions and their central axons survive ^6^. Multiple animal studies have shown that local delivery of neurotrophins, endogenous players in the signaling pathways involved in neuronal development and maintenance, can elicit neurite extension from spiral ganglion cells even in the adult mammalian ear ^72^. Several recent studies in the mouse and guinea pig have shown that at least within 24 hours post exposure, neurotrophin delivery can repair the noise-induced synaptic damage as it restores ABR amplitudes ^7, 73, 74^. Regeneration is likely more difficult at longer post-exposure times, but even in humans the distance from cell body to hair cell is <0.5 mm, and spiral ganglion cell death must be extremely slow because cochlear implants inserted years after deafness onset still provide useful hearing ^75^. Thus, it does not seem too far-fetched to imagine that there could be therapies for hidden hearing loss on the horizon.
